# Effects of Gegen Dingxuan Capsule on behavior, X-Ray Signs of the Cervical Spine, and Humoral Factor Levels in a Rat Model of Cervical Vertigo

**DOI:** 10.1155/2019/9524162

**Published:** 2019-07-22

**Authors:** Guang-kui Feng, Xian-jun Ma, Yin-yi Chen, Li Wang, Qain Du, Ru-yue Shi, Zi Meng

**Affiliations:** ^1^Department of Encephalopathy, Lianyungang Affiliated Hospital, Nanjing University of Chinese Medicine, Lianyungang 222004, China; ^2^Lianyungang Higher Vocational Technical College of Traditional Chinese Medicine, Lianyungang 222000, China

## Abstract

**Objective:**

To investigate the effects of Gegen Dingxuan capsule on behavior, X-ray signs of the cervical spine, and levels of norepinephrine (NE), nitric oxide (NO), endothelin (ET-1), and calcitonin gene-related peptide (CGRP) in the plasma of a rat model of cervical vertigo and additionally to clarify the underlying mechanisms of action.

**Method:**

A total of 40 male SPF Sprague-Dawley rats were randomly assigned to blank control, model, Sibelium, and Gegen Dingxuan capsule groups, with 10 rats in each group. A rat model of cervical vertigo was produced by physically damaging the cervical spine, thereby perturbing its stability. After cervical spine surgery, rats in the Sibelium and Gegen Dingxuan capsule groups were administered Sibelium and Gegen Dingxuan capsule, respectively. After 4 and 8 weeks of administration, balance beam test was used to assess behavior, lateral X-ray images of the cervical spine were taken and scored, and the plasma levels of NE, NO, ET-1, and CGRP were measured.

**Results:**

After 4 and 8 weeks of drug administration, the balance beam test scores in the Gegen Dingxuan capsule group were significantly higher than those in the Sibelium group. The radiographic scores were significantly lower in the Gegen Dingxuan capsule group than those in the Sibelium group at 8 weeks. Plasma NE, NO, ET-1 levels, and ET-1/CGRP ratio were significantly decreased in the Gegen Dingxuan capsule group compared with the model group. No significant difference was found between the Sibelium and Gegen Dingxuan capsule groups. Plasma CGRP levels were significantly increased in the Gegen Dingxuan capsule group compared with the model group and were significantly decreased compared with the Sibelium group.

**Conclusions:**

Gegen Dingxuan capsule improves behavior, radiographic scores, reduces plasma levels of NE, NO, ET-1, and the ET-1/CGRP ratio, and increases plasma CGRP levels. Gegen Dingxuan capsule may improve outcome in the rat model of cervical vertigo by ameliorating cervical facet joint disorder, relieving cervical muscle spasm and vasospasm, increasing blood supply, and regulating humoral factor levels.

## 1. Introduction

Cervical vertigo is characterized by vertigo originating from the cervical spine and is associated with many syndromes, including dizziness, which can be worsened by head motion, nausea and vomiting, gait unsteadiness, neck pain or stiffness, numbness, and tinnitus [1]. With the rapid development of intelligent mobile devices, lifestyles have undergone tremendous change, including a rapid increase in “phubbing”, and as a consequence, the prevalence of cervical vertigo has increased. Although the diagnosis of cervical vertigo is difficult, and there is a lack of epidemiological data, cervical vertigo has become one of the most frequently encountered diseases [2].

Gegen Dingxuan capsule, which is prepared in our hospital, was approved by the Jiangsu Drug Administration in 2004. Through 14 years of clinical practice, we have discovered that Gegen Dingxuan capsule has good clinical efficacy in improving cervical vertigo, and consequently it has become a routine treatment for the condition in our hospital. Gegen Dingxuan capsule effectively improves the symptoms of vertigo and neck pain or stiffness [3]. It takes effect rapidly and is associated with a low recurrence rate. In this study, we investigate the effects of Gegen Dingxuan capsule on behavior, X-ray signs of the cervical spine, and plasma levels of humoral factors, including norepinephrine (NE), nitric oxide (NO), endothelin (ET-1), and calcitonin gene-related peptide (CGRP) in a rat model of cervical vertigo, and we explore the underlying mechanisms of action.

## 2. Materials and Methods

### 2.1. Materials

Forty male Sprague-Dawley rats, SPF grade, weighing 200 ± 20 g, were provided by the Animal Experimental Center of Nanjing University of Chinese Medicine of China (license number: SCXK (Su) 2013-0003). After one week of acclimation, rats were randomly assigned to blank control, model, Sibelium, and Gegen Dingxuan capsule groups, with 10 rats in each group. All procedures were approved by the Animal Ethics Committee of Lianyungang Affiliated Hospital, Nanjing University of Chinese Medicine.

Gegen Dingxuan capsule was provided by the Manufacturing Laboratory, Lianyungang Affiliated Hospital, Nanjing University of Chinese Medicine, China. For drug preparation,* Radix puerariae, Ramulus Cinnamomi, Raw Radix Paeoniae Alba, Radix Glycyrrhizae preparata, Bombyx Batryticatus, Rhizoma Seu Radix Notopterygii, Rhizoma Chuanxiong, Ramulus Uncariae Cum Uncis, Rhizoma Zingiberis Recens, *and* Fructus Jujubae* were formulated at a ratio of 10:4:4:2:3.3:3.3:5:5:5. Half of the* Radix puerariae *and* Rhizoma Chuanxiong* were processed into fine powder, sifted through a sieve, and mixed thoroughly. The remaining herbs were decocted three times with water and filtered. The filtrates were mixed and concentrated. Ethanol was added to a final concentration of 60%. After standing, the ethanol was recovered. Decoctions were condensed under vacuum, mixed with the fine powder described above, and stored in bottles (No. Z04001243, 0.45 g × 36 capsules/bottle). Flunarizine hydrochloride capsules (Sibelium, No. H10930003, 5 mg/pill) were purchased from Xi'an Janssen Pharmaceutical (Xi'an, China).

The administration dose for the Gegen Dingxuan capsule group was 486 mg/kg (equivalent to the dose for adults [1.8 mg (4 capsules), three times a day]). The administration dose for the Sibelium group was 0.45 mg/kg (equivalent to the dose for adults [5 mg, once a day]).

Reagents included benzylpenicillin sodium for injection (0.96 g, 1.6 million units, North China Pharmaceutical Co., Ltd. China), chloral hydrate (AR grade, Sinopharm Chemical Reagent Co., Ltd., Shanghai, China), Rat ET enzyme-linked immunosorbent assay (ELISA) kit, Rat CGRP ELISA kit, and Rat NE ELISA kit. A DG5033A microplate reader (Nanjing Huadong Electronics Group Medical Equipment Co., Ltd., China) was used for the colorimetric assays, and a self-made wooden beam (2 cm × 80 cm) was used for the balance beam test.

### 2.2. Methods

All rats were given cervical spine surgery, except the blank control group. The rat model of cervical vertigo was produced using the cervical instability method [1], with modification to reduce intraoperative risk and trauma. Briefly, the posterior cervical muscles were excised, and the spinous process and ligaments, including the intertransverse and interspinous ligaments, were removed at the C5–C6 level. The C5–C6 joint capsule was obliterated with scissors, and the upper and lower vertebral bodies were damaged, resulting in cervical spine instability. The wound was then sutured layer by layer. Each rat received injection of penicillin (40,000 U/kg body weight) (Phoenix Scientific) after surgery. One week after surgery, the wound was healed, and rats were placed on the balance beam. The rat model of cervical vertigo was considered to have been successfully modeled if the rat met the following criteria: it was unable to stand or move on the beam, body shook on the beam, or it stopped, it changed direction abruptly and could not cross the beam. The medicine was intragastrically administered (1 ml/100 g, once a day) at 8:00–12:00 a.m. In the blank control and model groups, an equal volume of double distilled water was administered daily. After 4 and 8 weeks of administration, blood was collected from the orbit of the eye and placed in a centrifuge tube with anticoagulants. After thorough mixing, samples were stored at −80°C.

### 2.3. Balance Beam Test [2]

A self-made balance beam apparatus (2 cm × 80 cm) was suspended above the ground. After moving their head up and down, left and right, rats were place on the beam to assess their ability to traverse the wooden beam. Performance was rated according to a scale devised in our laboratory, as follows: No (0) point was given if the rat could not move on the wooden beam, if its body shook on the beam, or it stopped and changed direction abruptly, and moved slightly, but was unable to traverse the beam; 1.5 points were given if its body shook on the beam, it stopped and changed direction abruptly, but moved and traversed the beam; 3 points were given if the rat moved smoothly and traversed the beam. Performance was assessed at the beginning of the experiment and at 4 and 8 weeks after drug administration.

### 2.4. X-Ray Examination

Rats were anesthetized with injections of 1% sodium pentobarbital (40 mg/kg). Lateral view cervical spine X-rays were taken and scored according to the method of Song et al. [1], as follows.

(1) Physiological lordosis of the cervical spine is as follows: 0 = the presence of cervical physiological lordosis; 1 = straightening of cervical physiological lordosis; 2 = loss of cervical physiological lordosis; 3 = complete loss of cervical physiological lordosis.

(2) Disc space narrowing is as follows: 0 = normal; 1 = mild (10% narrowing); 2 = moderate (30% narrowing); 3 = severe (50% narrowing).

(3) Vertebral body osteophytes on the anterior and posterior edges of the vertebral bodies are as follows: 0  =  none; 1  =  mild osteophyte; 2  =  moderate osteophyte; 3 = large osteophyte.

(4) Sclerosis and osteophyte formation in the uncovertebral joints and facet joints is as follows: 0 = none; 1 = blurring of the joints; 2 = mild sclerosis and osteophyte formation; 3 = obvious sclerosis and osteophyte formation. The radiographic scores were recorded 8 weeks after drug administration.

### 2.5. Detection of Plasma ET-1 Levels

Plasma ET-1 levels were detected by ELISA, according to the manufacturer's instructions.

### 2.6. Detection of Plasma CGRP Levels

Plasma CGRP levels were detected using the double-antibody sandwich technique, according to the manufacturer's instructions.

### 2.7. Detection of Plasma NE Levels

Plasma NE levels were detected by ELISA. A 40-*μ*l volume of plasma was taken, and 10 *μ*l of antiluteinizing hormone (LH) antibody and 50 *μ*l of streptavidin-HRP were added [1]. All the procedures were performed in accordance with the manufacturer's instructions. After generating the standard curve, plasma NE levels were calculated.

### 2.8. Detection of Plasma NO Levels

Plasma NO levels were detected using the nitrate reductase method. A 100-*μ*l sample of plasma was placed in a test tube, and after thorough mixing, the solution was incubated in a water bath at 37°C and then allowed to stand at room temperature for 10 minutes. After centrifugation, the supernatant was collected. After color development, the supernatants were mixed and allowed to stand. The absorbance at 550 nm (adjusted to 0 with distilled water) was measured, and plasma NO levels were calculated.

### 2.9. Statistical Analysis

Data were analyzed using SPSS 19.0 software. Measurement data were expressed as the mean ± SD, and the* t*-test was used to evaluate differences between groups (two-tailed).* P* < 0.05 was considered statistically significant.* P* < 0.01 was considered remarkably statistically significant.

## 3. Results

### 3.1. Balance Beam Test

One week after surgery, prior to drug administration, the balance beam test scores were significantly lower in the model, Sibelium and Gegen Dingxuan capsule groups, compared with the blank control group (*P *< 0.01), with no significant difference among the three groups given surgery, indicating that the cervical vertigo model was successfully produced. After 4 and 8 weeks of drug administration, the balance beam test scores were significantly higher in the Sibelium and Gegen Dingxuan capsule groups compared with the model group (*P *< 0.01), and the Gegen Dingxuan capsule group had significantly higher scores than the Sibelium group (*P *< 0.01) (Table 1).

### 3.2. Lateral View Cervical Spine X-Ray Scores

The model group had significantly higher radiographic scores than the blank control group (*P *< 0.01), indicating that model of cervical vertigo was successfully produced. After 8 weeks of administration, the radiographic scores in the Sibelium and Gegen Dingxuan capsule groups were both significantly lower than those in the blank control and model groups (*P *< 0.01). Furthermore, the Gegen Dingxuan capsule group had significantly lower radiographic scores than the Sibelium group (*P *< 0.01) (Table 2).

### 3.3. Plasma ET-1 Levels

Compared with the blank control group, plasma ET-1 levels were significantly increased in the model group (*P *< 0.01). Compared with the blank control and model groups, plasma ET-1 levels were significantly decreased in the Sibelium and Gegen Dingxuan capsule groups at 4 weeks (*P *< 0.01) and were further decreased at 8 weeks of administration (*P *< 0.01). No significant difference was found between the Sibelium and Gegen Dingxuan capsule groups (*P *> 0.05) (Table 3).

### 3.4. Plasma CGRP Levels

Plasma CGRP levels in the model group were significantly decreased compared with the blank control group (*P *< 0.01). Plasma CGRP levels were significantly increased in the Sibelium and Gegen Dingxuan capsule groups at 4 weeks (*P *< 0.01) and were further increased at 8 weeks of drug administration (*P *< 0.01). Furthermore, plasma CGRP levels were significantly decreased in the Gegen Dingxuan capsule group compared with the Sibelium group (*P *< 0.05) (Table 4).

### 3.5. Plasma ET-1/CGRP Ratio

In comparison with the blank control group, the plasma ET-1/CGRP ratio was significantly increased in the model group (*P *< 0.01). Compared with the model group, the plasma ET-1/CGRP ratio was significantly decreased in the Sibelium and Gegen Dingxuan capsule groups at 4 weeks (*P *< 0.01) and were further decreased at 8 weeks of drug administration (*P *< 0.01). There was no significant difference between the Sibelium and Gegen Dingxuan capsule groups (*P *> 0.05) (Table 5).

### 3.6. Plasma NE Levels

Plasma NE levels were increased in the model group. Plasma NE levels were significantly decreased in the Sibelium and Gegen Dingxuan capsule groups compared with the model group at 4 weeks (*P *< 0.01) and were further decreased at 8 weeks compared with the blank control and model groups (*P *< 0.01). There was no significant difference between the Sibelium and Gegen Dingxuan capsule groups (Table 6).

### 3.7. Plasma NO Levels

Plasma NO levels were increased in the model group. Plasma NO levels were significantly decreased in the Sibelium and Gegen Dingxuan capsule groups at 4 weeks compared with the model group (*P *< 0.01) and were further decreased at 8 weeks compared with the blank control and model groups (*P *< 0.01). No significant difference was found between the Sibelium and Gegen Dingxuan capsule groups (Table 7).

### 3.8. Discussion

Cervical vertigo is a controversial subject, and there are many hypotheses on its pathogenesis. Some investigators have posited that cervical vertigo is related to the straightening of the cervical spine or cervical kyphosis, resulting in instability of the cervical spine under physiological load [3, 4]. This, in turn, can trigger cervical sympathetic excitation, causing spasms of the vertebral basal artery, thereby producing cervical vertigo. In the present study, a rat model of cervical vertigo was produced by perturbing the stability of the cervical spine, according to the method of Song et al. [1]. The surgical procedure is simple and has a high success rate [5]. The model conforms with the underlying rationale of the disease and can be used as an ideal animal model of cervical vertigo. Flunarizine hydrochloride (Sibelium) is a commonly used drug for the treatment of vertigo. It prevents vasospasm of the vertebral basilar artery [6]. Therefore, Sibelium was used as a positive control drug in this study.

Cervical vertigo belongs to the category of vertigo and cervical spondylopathy according to traditional Chinese medicine. Cervical vertigo is mainly caused by overstrain, (neck) tendon injury, loss of the warming function of* Qi*, collateral vein obstruction, as well as by internal and external cold and dampness-induced stagnation of the tendons and veins. As a result,* Qi* and blood are unable to sufficiently nourish the seven apertures in the human head. Thus, cold dampness and astringency, discomfort of muscles and veins, and dysfunction of* Qi* and blood are the key pathological features of cervical vertigo. Treatment is needed to relax the muscles and tendons and to promote the* taiyang* meridian. Gegen Dingxuan capsule is an in-hospital preparation approved by our hospital in 2004. Gegen Dingxuan capsule consists of* Ramuli Cinnamomi *decoction plus* Radix puerariae, Rhizoma Seu Radix Notopterygii, Rhizoma Chuanxiong, Bombyx Batryticatus, *and* Ramulus Uncariae Cum Uncis*. This prescription focuses on relieving muscles, relaxing tendons and dredging collaterals, and promoting the* taiyang* meridian. The* Treatise on Febrile Diseases* points out that* Ramuli Cinnamomi *decoction plus* Radix puerariae* can be used for* taiyang* disease, stiff nape and back, sweating and fear of wind.* Ramuli Cinnamomi* decoction plus* Radix puerariae* is mainly used for treating diseases caused by exogenous pathogenic factors. It is inappropriate for the syndrome of fatigue and internal injury. However,* Ramuli Cinnamomi* decoction plus Radix puerariae regulates nutrition and nourishes* wei*, can be used to treat diseases caused by exogenous pathogenic factors or internal injury, and can relieve muscles and* ying*. Among the constituents in Gegen Dingxuan capsule,* Ramuli Cinnamomi* decoction plus* Radix puerariae* promotes meridians and relieves muscles and* ying*.* Rhizoma Seu Radix Notopterygii* is pungent in flavor and warm in property, dredges collaterals, dissipates cold, and dispels dampness.* Rhizoma Chuanxiong* dredges collaterals and activates blood circulation.* Bombyx Batryticatus* eliminates wind and activates collaterals.* Ramulus Uncariae Cum Uncis* stops endogenous wind and dredges collaterals. Together, the bioactive ingredients in Gegen Dingxuan capsule relieve muscles, dredge collaterals, promote blood circulation, and relax meridians to relieve vertigo and other symptoms.

In the current study, behavioral scores were improved in the Sibelium and Gegen Dingxuan capsule groups at 4 and 8 weeks of drug administration, compared with the model group, with Gegen Dingxuan capsule having a better effect than Sibelium at the latter time point. This result is in agreement with the radiographic scores, which were improved in the Sibelium and Gegen Dingxuan capsule groups at 8 weeks, with the Gegen Dingxuan capsule group having a lower radiographic score than the Sibelium group.

Cinnamaldehyde has been shown to improve muscle spasms and contraction [7]. Paeoniflorin and glycyrrhizic acid relieve muscle spasm and exert analgesic effects [8]. Ammonium oxalate in* Bombyx Batryticatus* has an anticonvulsant effect and relieves muscle tension and stiffness [9]. Therefore, Gegen Dingxuan capsule may improve behavior and cervical stability not only by acting on blood vessels, but also by alleviating cervical muscle spasm and improving cervical sympathetic excitability.

The cervical sympathetic nerves are widely distributed within the cervical vertebrae, intervertebral discs, cervical muscle tissue, and the adventitia of the vertebrobasilar artery. The sympathetic nerves distributed on the vertebral artery control blood flow. When the sympathetic nerves are stimulated, spasm of the vertebral arteries occurs, which can lead to vertebrobasilar artery ischemia and symptoms of vertigo. The symptoms of vertigo vary and are dependent on changes in sympathetic nervous system activity [10]. Furthermore, the sympathetic nervous system can induce emotional problems [11]. Lesions of the cervical spine can cause excitation of the sympathetic nerves of the vertebrobasilar artery, leading to spasm of the artery and posterior circulatory ischemia, resulting in dizziness and vertigo. A study [11] showed that the symptoms in patients with cervical vertigo are closely related to pain, stiffness, and discomfort in the cervical spine. Vertigo is aggravated if the cervical pain, stiffness, and discomfort are severe, which can be alleviated by improving cervical symptoms. NE is an indicator of sympathetic activation and can induce vasoconstriction by stimulating the *α*1 receptor in blood vessels. A decrease in NE levels indicates that abnormal sympathetic activity is controlled and that vasoconstriction is reduced. In the present study, we found that plasma NE levels in the Sibelium and Gegen Dingxuan capsule groups were both significantly decreased compared with the model group at 4 weeks and were further decreased at 8 weeks, with both drugs having a similar effect. This result indicates that the effectiveness of Sibelium and Gegen Dingxuan capsule both increase with time.

Pharmacological studies show that puerarin in* Radix puerariae* improves microcirculation, increases local blood flow, and inhibits platelet aggregation [12]. The active components in* Rhizomza Seu Radix Notopterygii* increase blood volume when local organ ischemia occurs [13]. The bioactive component in* Rhizoma Chuanxiong*, ligustrazine, inhibits endothelin production, reduces vascular resistance, protects vascular endothelial cells from damage, reduces platelet surface activity, and inhibits platelet aggregation [14].* Bombyx Batryticatus* also promotes microcirculation. The decoction of* Bombyx Batryticatus* increases the number of open capillaries, increases the diameter of microvessels, and prolongs clotting time [15]. The effect of Chinese medicines on NE levels has not been reported. Here, we found that Gegen Dingxuan capsule reduces NE levels, suggesting that it improves cervical vertigo by reducing plasma NE levels and modulating blood circulation.

Humoral factors, such as NE, ET-1, CGRP, and NO, may play important roles in the pathogenesis of cervical vertigo [16, 17]. NO has a vasodilatory effect and regulates the tension in blood vessels together with vasoconstrictors. When tissues are insufficiently perfused, vasoconstriction is induced by stress to raise perfusion pressure. At the same time, the release of NO dilates blood vessels, reducing resistance to blood flow, thereby increasing blood supply, especially during acute cerebral ischemia. In this study, plasma NO levels were significantly higher in the model group compared with the blank control group, in contrast to a previous report [18]. This discrepancy may be caused by differences in the animal models used. Here, spasm of the vertebral arteries in rats in the model group may cause a compensatory increase in NO levels, and the unstable vertebrae may continuously stimulate the surrounding tissue to produce an inflammatory response, resulting in a significant increase in inducible nitric oxide synthase (iNOS) levels. Indeed, iNOS expressed in activated microglia and glial cells can produce a large amount of NO under pathological conditions such as inflammation and cerebral ischemia [19]. We speculate that an inflammatory response is induced by cervical spine injury in rats, leading to excessive production of NO. After 4 weeks of drug administration, the plasma NO levels in the Gegen Dingxuan capsule group were significantly decreased compared with the model group. This reduction might be associated with an improvement in stress-triggered vasoconstriction and the inhibition of NO production. A study [20] showed that Guizhi plus Gegen Decoction helps prevent intervertebral disc degeneration caused by annulus fibrosus cell apoptosis, perhaps by regulating NO levels. Furthermore, Guizhi plus Gegen Decoction protects against LPS-induced cortical neuronal injury in rats with neuroinflammation. The underlying mechanism may be associated with inhibition of cortical microglial activation and the regulation of proinflammatory cytokines, such as tumor necrosis factor alpha (TNF-*α*) and interleukin-1*β* (IL-1*β*) [21]. Inhibition of TNF-*α* and IL-1*β* release can suppress the production of iNOS. Flavonoids from* Radix puerariae* have been shown to regulate NO content in rats [22].* Rhizoma Chuanxiong* can downregulate NF-*κ*B, inhibit iNOS activity, and reduce iNOS levels [23]. The anti-inflammatory effect of total glucosides of* Paeoniae Radix Alba* is closely related to the inhibition of NF-*κ*B, reduction of iNOS expression, and NO production in macrophages [24]. Rhynchophylline can reduce NO production, inhibit NOS activity in the brain, suppress the formation of free radicals, and exert a neuroprotective effect [25]. Based on these previous observations and our current findings, we speculate that Gegen Dingxuan capsule improves cervical vertigo by regulating the expression of iNOS, thereby reducing NO levels and exerting anti-inflammatory and analgesic effects, and inhibiting sympathetic excitation. While no significant difference in plasma NO was found between the Sibelium and Gegen Dingxuan capsule groups, levels were lower in the Gegen Dingxuan capsule group. Although Sibelium may reduce vasospasm, it may not exert anti-inflammatory or analgesic effects because of an inability to regulate iNOS, in contrast to Gegen Dingxuan capsule.

ET and CGRP are among the most powerful endogenous vasoconstrictors and vasodilators [26]. ET and CGRP work together to regulate blood flow and maintain vascular function. Studies suggest that plasma ET-1 and CGRP are key pathogenetic factors in cervical vertigo and that an imbalance of ET-1 and CGRP (i.e., a perturbation of the ET-1/CGRP ratio) is closely related to the development of cervical vertigo [17, 27]. This is in contrast to NE, which is involved in sympathetic activities [28]. In the present study, we found that after 4 and 8 weeks of drug administration, the plasma ET-1 levels and ET-1/CGRP ratio were significantly decreased in the Sibelium and Gegen Dingxuan capsule groups, to similar levels, compared with the blank control and model groups. The plasma CGRP levels were significantly increased in the Sibelium and Gegen Dingxuan capsule groups compared with the blank control and model groups. It is unclear whether the changes in humoral factors are the cause or result of vertigo, but we speculate that, in our rat model, the perturbation in humoral factors might result from cervical vertigo. It has been reported that the main components of* Radix puerariae*, flavonoids, dilate blood vessels in the brain and inner ear and regulate the relative plasma levels of ET-1 and CGPR [29]. Ferulic acid, the active component of* Rhizoma Chuanxiong*, can inhibit ET and its receptors [30].

There are some limitations to this study. While conventional doses of Gegen Dingxuan capsule were used, we did not evaluate the effects of higher or lower doses. In a future study, we will examine the dose–effect relationship of Gegen Dingxuan capsule on cervical vertigo. Additionally, we did not examine vertebrobasilar blood supply, but will do so in a future study using the transcranial Doppler technique.

## 4. Conclusion

Our findings show that Gegen Dingxuan capsule improves behavior, radiographic scores, reduces plasma levels of NE, NO, ET-1, and the ET-1/CGRP ratio, and increases plasma CGRP levels in a rat model of cervical vertigo. The therapeutic mechanism of action of Gegen Dingxuan capsule may be associated with an improvement in cervical facet joint disorder, reduction of vasospasm of the vertebrobasilar artery, and an increase in blood supply.

## Figures and Tables

**Table 1:** Comparison of balance beam scores before and after drug administration (mean ± SD).

Groups	Number	After rat model establishment	4 weeks after administration	8 weeks after administration
Blank control group	10	3.00 ± 0.00	3.00 ± 0.00	3.00 ± 0.00
Model group	10	0.30 ± 0.60^▲^	0.30 ± 0.60^▲^	0.15 ± 0.45^▲^
Sibelium group	10	0.15 ± 0.45^▲^	0.9 ± 0.77^▲^	1.35 ± 0.81^▲△^
Gegen Dingxuan capsule group	10	0.15 ± 0.45^▲^	1.65 ± 0.81^▲●^	2.4 ± 0.73^*∗*△#^

^▲^ *p*< 0.01, *∗p* < 0.05 vs. blank control group; ^●^*p* < 0.05, ^△^*p* < 0.01 vs. model group; ^#^*p* < 0.01 vs. Sibelium group.

**Table 2:** Comparison of radiographic scores after 8 weeks of drug administration (*n* = 10, mean ± SD).

Groups	Administration dosages (mg/kg)	X-Ray examination
Blank control group	-	0.00 ± 0.00
Model group		7.40 ± 0.97^▲▲^
Sibelium group	0.45	4.50 ± 1.27^▲▲*∗∗*^
Gegen Dingxuan capsule group	486	2.50 ± 1.18^▲▲*∗∗*##^

^▲▲^ *p* < 0.01 vs. blank control group; ^*∗∗*^*p* < 0.01 vs. model group; ^##^*p* < 0.01 vs. Sibelium group.

**Table 3:** Comparison of plasma ET-1 levels after 4 and 8 weeks of drug administration (*n* = 10, mean ± SD).

Groups	Administration	4 weeks after	8 weeks after
	dosages (mg/kg)	administration (pg/ml)	administration (pg/ml)
Blank control	-	54.72 ± 13.85	54.84 ± 11.91
Model group	-	143.34 ± 15.66^▲▲^	135.22 ± 14.46^▲▲^
Sibelium group	0.45	87.11 ± 10.33^▲▲*∗∗*^	65.20 ± 8.73^▲*∗∗*^
Gegen Dingxuan capsule group	486	87.62 ± 13.51^▲▲*∗∗*^	66.39 ± 8.72^▲*∗∗*^

^▲▲^ *p* < 0.01, ^▲^*p* < 0.05 vs. blank control group; *∗∗p* < 0.01 vs. model group.

**Table 4:** Comparison of plasma CGRP levels after 4 and 8 weeks of drug administration (*n* = 10, mean ± SD).

Groups	Administration	4 weeks after	8 weeks after
	dosages (mg/kg)	administration (pg/ml)	administration (pg/ml)
Blank control	-	28.55 ± 3.16	29.10 ± 2.58
Model group	-	16.32 ± 2.96^▲▲^	16.45 ± 2.11^▲▲^
Sibelium group	0.45	25.15 ± 2.43^▲▲*∗∗*^	28.25 ± 1.86*∗∗*
Gegen Dingxuan capsule group	486	22.62 ± 1.91^▲*∗∗*#^	26.08 ± 1.68^▲*∗∗*#^

^▲▲^ *p* < 0.01, ^▲^*p* < 0.05 vs. blank control group; *∗∗p* < 0.01 vs. model group; and ^#^*p* < 0.05 vs. Sibelium group.

**Table 5:** Comparison of the plasma ET-1/CGRP ratios after 4 and 8 weeks of drug administration (*n* = 10, mean ± SD).

Groups	Administration dosages (mg/kg)	4 weeks after administration	8 weeks after administration (pg/ml)
Blank control group	-	1.92 ± 0.48	1.90 ± 0.44
Model group	-	9.05 ± 1.92^▲▲^	8.38 ± 1.67^▲▲^
Sibelium group	0.45	3.48 ± 0.46^▲▲*∗∗*^	2.31 ± 0.30*∗∗*
Gegen Dingxuan capsule group	486	3.87 ± 0.49^▲▲*∗∗*^	2.55 ± 0.32*∗∗*

^▲▲^ *p* < 0.01 vs. blank control group; *∗∗p* < 0.01 vs. model group.

**Table 6:** Comparison of the plasma NE levels after 4 and 8 weeks of drug administration (ng/ml, mean ± SD).

Groups	Number	4 weeks after administration	8 weeks after administration
Blank control group	10	34.40 ± 5.52	35.67 ± 493
Model group	10	77.28 ± 8.16	75.34 ± 7.37
Sibelium group	10	47.94 ± 7.24^*∗*^	42.98 ± 7.28^#^
Gegen Dingxuan capsule group	10	42.56 ± 5.11^*∗*^	41.74 ± 5.63^#^

*∗p* < 0.01 vs. the model group; ^#^*p* < 0.01 vs. the blank control and model groups.

**Table 7:** Comparison of plasma NO levels after 4 and 8 weeks of drug administration (ng/ml, mean ± SD).

Groups	Number	4 weeks after administration	8 weeks after administration
Blank control group	10	34.88 ± 5.57	34.55 ± 5.48
Model group	10	80.26 ± 10.95	82.50 ± 13.18
Sibelium group	10	47.01 ± 8.43^*∗*^	43.38 ± 7.45^*∗*^
Gegen Dingxuan capsule group	10	44.16 ± 8.05^*∗*^	42.05 ± 6.71^*∗*△^

*∗p* < 0.01 vs. the model group; ^△^*p* < 0.05 vs. the blank control group.

## Data Availability

Medicine is the achievement of our independent research. Data and figures in it are real without any fabrication. In addition to the content quoted through the annotated references, this manuscript does not contain research results which have been published or written by any other person. All the individuals and groups making an important contribution to the study of this manuscript have been marked in a clear manner. We are willing to undertake all the legal consequences of the data or conclusions of this manuscript. The data used to support the findings of this study are included within the article.
